# Does Microbiome Matter in Chronic Intestinal Failure Due to Type 1 Short Bowel Syndrome in Adults?

**DOI:** 10.3390/nu16142282

**Published:** 2024-07-16

**Authors:** Marta Ossola, Ilario Ferrocino, Irene Franciosa, Umberto Aimasso, Leila Cravero, Ambra Bonciolini, Vladimiro Cardenia, Fabio Dario Merlo, Marta Anrò, Alessia Chiarotto, Clara Bosa, Luca Cocolin, Simona Bo

**Affiliations:** 1 Dietetic and Clinical Nutrition Unit, Città della Salute e della Scienza Hospital, C.so Bramante 88, 10126 Torino, Italy; 2 Department of Agricultural, Forest and Food Sciences, University of Turin, Largo Paolo Braccini 2, Grugliasco, 10095 Torino, Italy; 3 Department of Medical Science, University of Torino, C.so Dogliotti 14, 10126 Torino, Italy

**Keywords:** chronic intestinal failure, metabolome, short chain fatty acids, small bowel syndrome, volatile organic compounds

## Abstract

The exact microbiome composition and function of patients with Short Bowel Syndrome (SBS) and Chronic Intestinal Failure (CIF) are still unknown. Patients with type I SBS-CIF (end-jejunostomy/ileostomy) are little represented in available studies. The aim of this study is to evaluate the microbiome characteristics of adult type 1 SBS-CIF patients according to their clinical features. Fecal microbiota was studied by amplicon-based sequencing and volatile organic compounds (VOCs) were assessed by solid-phase microextraction and gas chromatography–mass spectrometry. A total of 44 adult type 1 SBS-CIF patients were enrolled. At the family level, *Lactobacillaceae* (38% of the relative frequency) and *Streptococcaceae* (24%) were predominant; at the genus level, *Streptococcus* (38% of the relative frequency) and *Lactobacillus* (24%) were the dominant amplicon sequence variants (ASVs). Patients with increased stomal output showed higher ASVs for *Lactobacillus* (Rho = +0.38; *p* = 0.010), which was confirmed after adjusting for small bowel length (OR = 1.04; 95% CI 1.01–1.07, *p* = 0.023). Hyperphagia was associated with higher concentrations of short-chain fatty acid (SCFA) esters, such as butanoic acid ethyl ester (*p* = 0.005) and hexanoic acid ethyl ester (*p* = 0.004). Dietary fiber intake was directly correlated with most VOCs. Hyperphagia was associated with dietary fiber, after adjusting for small bowel length (OR = 1.35; 95% CI 1.01–1.81; *p* = 0.040). In type 1 SBS-CIF patients, a greater frequency of *Lactobacilli* was associated with increased stomal outputs, while increased fiber intake and concentrations of SCFA esters were associated with hyperphagia. These results might have implications for clinical practice.

## 1. Introduction

Chronic Intestinal Failure (CIF) is the long-lasting reduction in gut function below the minimum necessary for the absorption of macronutrients and/or water and electrolytes; as a result, intravenous supplementation is required to maintain health and nutritional homeostasis [1]. The most common cause of CIF is Short Bowel Syndrome (SBS), a rare condition defined by a remaining small intestinal length in continuity of <200 cm as measured from the duodenojejunal junction because of surgical resections for various diseases [2]. Three main anatomical SBS types can be distinguished based on the site of resection: type 1, or the end-jejunostomy or end-ileostomy, resulting from the resection of both the colon and the ileum (total or partial); type 2, or jejuno-colonic anastomosis, resulting from the resection of all or most of the ileum and part of the colon; and type 3, or jejuno-ileal anastomosis, which is when a portion of the terminal ileum is in continuity with an intact colon with preservation of the ileocecal valve [3].

The gut microbiota plays a key physiological role in different gastrointestinal functions, such as food digestion and nutrient metabolism, micronutrient synthesis, hunger–satiety control, immune system and metabolism regulation, barrier function, protection against pathogens, fermentation and gas production, compound detoxification, and gut motility [4]. Its importance on the prognosis of patients with SBS was assumed a few years ago since the first animal studies documented a relevant dysbiosis after small bowel resection, which was associated with increased colonic mucosa inflammation and SBS-related liver disease [5,6,7]. A few human studies evaluated the gut microbiota composition of SBS adults and reported an overall reduction in microbial diversity (α-diversity) and bacterial richness, and a microbial imbalance [8,9,10,11,12]. Despite differences in the underlying diseases and clinical situations, a very similar pattern of gut microbiota dysbiosis was described among adult patients, with a high prevalence of *Lactobacilli* together with a depletion in nonpathogenic *Clostridia*, a reduction in commensal bacteria from *Lachnospiraceae*, *Ruminococcaceae*, and *Bacteroidaceae*, a paucity of anaerobes, and an abundance of *Enterobacteriaceae* compared with healthy subjects [9,10,11,12,13,14]. Similar findings were reported in children, as recently reviewed in [15]. In patients with CIF, gut microbial dysbiosis has been related to unfavorable outcomes, such as liver disease, D-lactic acidosis, increased duration of intestinal adaptation, reduced weaning capacity, and in children, poor growth [15]. On the other hand, gut microorganisms have been reported to also display a beneficial role with energy savage, and adaptive mechanisms after gut resection in SBS patients [11].

Previous adult human studies evaluated SBS patients with highly different anatomic conditions, including patients with or without a colon in continuity, with or without the ileocecal valve, with or without ostomy, and the number of included type 1 SBS-CIF patients ranged from 0 [8,9,10] to 10 [13] and 9 [14]. It is well known that the colon in continuity favorably impacts the adaptation processes due to gastrointestinal hormones and the production of short-chain fatty acids (SCFAs) by the prominent anaerobic microbiota, and that the presence of the ileocecal valve influences the gut microbiota composition by creating a different intestinal microenvironment [3,10,16]. Furthermore, patients with a terminal jejunostomy have the greatest need for parenteral supply, are less likely to undergo intestinal adaptation, and show a greater number of complications, determining higher healthcare costs [2,17,18].

Finally, assessment of the production of metabolites by the microbiota may shed light on the functional role of resident microbiota. To date, metabolomic analyses have been conducted in animals [7,19,20,21], in a small number of pediatric patients [22,23], and in two adult studies that were not specifically focused on type 1 SBS-CIF patients, with the number of these latter individuals being ≤10 [13,14]. In the small bowel, bacterial fermentation of carbohydrates and lipid catabolism lead to the production of gases, organic acids, alcohols, and aldehydes and are currently very little studied.

Owing to the paucity of available data, we aimed to assess the composition and characteristics of the gut microbiome in adult type 1 SBS-CIF patients as well as the possible differences according to their clinical features.

## 2. Materials and Methods

### 2.1. Participants

All the adult patients with a diagnosis of type 1 SBS and requiring parenteral nutrition (PN) were enrolled at the Intestinal Failure Unit of the “Città della Salute e della Scienza” Hospital of Torino, a tertiary referral center for CIF support. The enrolment period was from December 2022 to May 2023 (Supplementary Figure S1). The inclusion criteria were (i) age ≥ 18 years; (ii) a diagnosis of type 1 SBS confirmed by radiological examination; and (iii) need for PN > 3 months. All patients were on anti-secretory drugs (pantoprazole or lansoprazole) and antidiarrheal medication (loperamide) at the maximum dosage tolerated and were given a diet restricted in fiber and lactose, with an energy amount appropriate for the individual needs and corrected for the estimated fecal energy losses.

Exclusion criteria were active neoplastic disease and/or being under antineoplastic treatment within the previous 5 years; inability to give informed consent; critically ill patients or patients with a <6-month life expectancy; CIF-associated liver disease; active inflammatory bowel disease; use of antibiotic, prebiotic, probiotic, or postbiotic in the last 2 months; treatment with glucagon-like-peptide agonists, steroids, or immunosuppressive drugs in the last 6 months; reconstructive surgery or intestinal transplantation, and pregnancy.

Forty-five patients met the criteria listed above, and one of them was uncooperative; thus, 44 patients were studied (Supplementary Figure S1).

### 2.2. Ethical Aspects

All patients gave informed written consent to participate in this study. This study was approved by the local Ethics Committee (protocol number 75802/2022); all the procedures were in accordance with the Declaration of Helsinki.

### 2.3. Procedures

Patients were asked to void their ostomy bag at 24:00 at home and fast for at least 10 h but not more than 12 h the day before the fecal sampling. Patients went to our center at 8:00 for stool collection. Fecal samples were obtained by emptying the content of the ostomy bag into a sterile container and taking a sample after mixing the stool with sterile gloves. Fecal samples were then transferred into a smaller sterile container (VWR, Milan, Italy) and stored at −80 °C for DNA extraction. No storage medium was used.

Body weight and height were measured at fasting in the morning, with the patients wearing light clothes and no shoes, respectively, by a mechanical column scale (SECA model 711) and a Stadiometer (SECA 220 measuring rod, Hamburg, Germany), after urination and emptying of the stoma bags.

Patients received follow-up at the Intestinal Failure Unit from their diagnosis of CIF until weaning from PN or death [24]. All laboratory and radiological examinations, anthropometric values, and clinical data were carefully recorded in the database of the unit. All patients received personalized nutritional and fluid support according to their individualized needs, considering body weight, 24 h urine, and ostomy output, oral caloric intakes, and laboratory exams in line with a standardized procedure by a team of trained physicians and registered dieticians. Nobody was on tube-enteral feeding. PN was administered at night by an intermittent schedule for 10–16 h/day.

A diet restricted in fiber and lactose, with an energy amount appropriate for the individual needs and corrected for the estimated fecal energy losses, was prescribed to all patients. Management of central venous catheters and PN complications, follow-up, and centralized laboratory and radiologic examinations were performed according to guidelines [1]. Monthly (or more frequently, if needed) domiciliary visits by specialized nurses guaranteed home care. Hospital follow-up visits were scheduled around every month.

### 2.4. Definitions

The same trained radiologist performed a barium or Gastrografin follow-through examination to estimate the residual intestinal length in all patients. SBS was diagnosed in the presence of a remnant small bowel length ≤ 200 cm [1]. Energy intakes were assessed during each visit by the same trained dietician by asking the frequency and portions of all the foods consumed in the last 7 days before the visit. Food intake was calculated as the ratio of oral energy intake (kcal/day) to resting energy expenditure (REE) (kcal/day) (Food Intake Ratio, FIR). REE was calculated for each patient using the Harris–Benedict equation. Hyperphagia was defined in the presence of an FIR score > 1.5, in line with the literature [25].

### 2.5. Volatilome Analysis

The volatile organic compounds (VOCs) in stool samples were extracted and analyzed using headspace (HS) solid-phase microextraction (SPME) coupled with gas chromatography–mass spectrometry (GC/MS). The GC-MS analyses were carried out by using a GC-2010 gas chromatograph equipped with a QP-2010 Plus quadruple mass spectrometer (Shimadzu Corporation, Kyoto, Japan), interfaced with a computerized system for data acquisition (Software GC–MS Solution V. 2.5, Shimadzu, Japan) and an RTX-Wax capillary column measuring 30 m in length, 0.25 mm in internal diameter, and 0.25 μm in film thickness (Restek, Bellefonte, PA, USA). Briefly, 100 mg of stool sample was collected into 20 mL vials and 10 µL 1-octanol was added as an internal standard for the compound semi-quantification (final concentration 350 ppm). Before starting the analyses, the fiber was conditioned into a GC inlet for 30 min (260 °C). Samples were equilibrated at 40 °C for 10 min and SPME fiber was exposed to the headspace for 40 min. The fiber was then inserted into the injection port (250 °C) of the gas chromatograph for 5 min for sample desorption. Helium with a constant flow of 1 mL/min was used as carrier gas. The temperature program was 40 °C for 1 min, followed by increments at the rate of 5 °C/min to 220 °C, then 10 °C/min to 250 °C, and lastly 300 °C for 20 min. The ion source and transfer line temperatures were set at 200 °C and 300 °C, respectively. Ions generated by election ionization (EI; 70 eV) were acquired in the range of 33–450 amu (1666 amu/s) as total ion current (TIC). The VOCs were recognized by comparing their mass spectra with those reported in the NIST08s (National Institute of Standards and Technology, Gaithersburg, MD, USA) library. According to Traina et al. [26], semi-quantitative data (mg/kg) were obtained by the internal standard method and measuring the TIC peak area of each identified compound.

All samples were analyzed in duplicate. VOCs in traces with less than 0.5 ppm concentration in at least two samples were excluded to increase the confidence of analyses.

### 2.6. DNA Extraction and Metagenomic Sequencing

The total DNA was extracted from all the collected stool samples using the QIAamp PowerFecal Pro DNA Kit following the manufacturer’s instructions. The extracted DNA was standardized at 100 ng/L (NanoDrop 1000 spectrophotometer; Thermo Scientific, Milan, Italy) and used to study the microbiota. The metataxonomic analyses were based on the V3–V4 region of the 16S rRNA gene of bacteria using primers and conditions previously reported [27]. PCR clean-up was performed with KAPA Pure Beads (Roche, Milan, Italy) and index PCR was performed with Illumina sequencing adapters using the Nextera XT Index Kit (Illumina, Milan, Italy) according to the 16S Metagenomic Sequencing Library Preparation (https://support.illumina.com/documents/documentation/chemistry_documentation/16s/16s-metagenomic-library-prep-guide-15044223-b.pdf, accessed on 1 May 2024). After the second clean-up, library normalization pooling was then performed. A MiSeq Illumina instrument (Illumina, Milan, Italy) with V3 chemistry was used to perform the sequencing by generating 2 × 250 bp paired-end reads.

### 2.7. Bioinformatic Analyses

The raw files (.*fastq*) generated during the sequencing were elaborated by QIIME 2 software [28]. The primer sequences were removed by Cutapter and DADA2 algorithms were used to denoise the obtained reads by using the q2-dada2 plugin in QIIME 2 [29] using the following parameters: --p-trunc-len-f 233 --p-trunc-len-r 229 --p-trunc-q 20 --p-max-ee-f 2 --p-max-ee-r 2 --p-chimera-method pooled. The QIIME2 feature classifier was used for taxonomy classification against the SILVA database. The amplicon sequence variants (ASVs) filtered at 0.5% in at least two samples were then used for downstream analysis.

### 2.8. Statistical Analyses

The results were reported as mean ± SD, median and interquartile range, or percentages, and differences between groups were analyzed by the *t*-Student’s test or the chi-square test. Correlations were calculated by Pearson or Spearman tests, as appropriate. Alpha diversity indexes (Shannon index, number of observations, and ACE index) were calculated by the diversity script of QIIME2.

The metabolome and microbiota data were analyzed using the Kruskal–Wallis or Wilcoxon test, as appropriate, as a function of the clinical variables in R-Studio, version 4.3.0. The VOC table was imported in R to build the heat plot by using the ade4 package in R-Studio. Multiple regression analyses were employed to evaluate the associations between clinical features and microbiome and volatilome characteristics of the patients.

## 3. Results

### 3.1. Characteristics of the Participants

Table 1 displays the participant’s characteristics. The most frequent underlying condition was inflammatory bowel disease, and women were the slight majority. Most patients had a high stomal output and were on long-lasting PN, with a low-grade chronic inflammatory state. In 16% of the cases, hyperphagia was present. Intakes of macronutrients expressed as a percentage of total calories were 49.9 ± 7.5 (carbohydrates), 16.9 ± 9.7 (sugars), 33.8 ± 5.1 (lipids), and 16.1 ± 3.0 (proteins); fibers were 9.8 ± 4.6 g/day.

### 3.2. Microbiome and Volatilome Characteristics of SBS-CIF Patients

After sequencing and denoising, a total of 1,262,682 reads were obtained with a mean of 28,697 reads/sample and a sample coverage > 99%.

**Figure 1:**
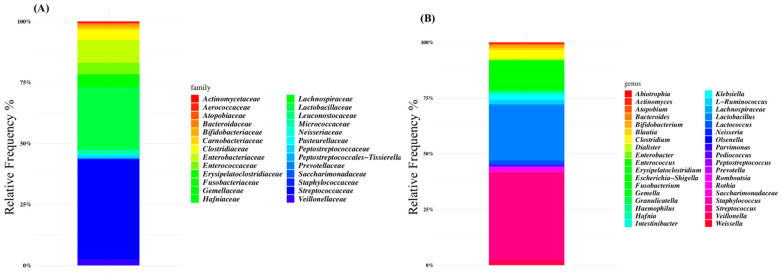
Global composition of gut microbiota of SBS-CIF patients at family (**A**) and genus levels (**B**). Only amplicon sequence variants (ASVs) with a relative frequency > 1% in at least 10% of subjects are shown.

The microbiota composition of patients was characterized at the family level by *Lactobacillaceae* (38% of the relative frequency), *Streptococcaceae* (24%), *Enterobacteriaceae* (7%), and *Enterococcaceae* (4%) (Figure 1A). At the genus level, *Streptococcus* was the dominant ASV (38% of the relative frequency on average), followed by *Lactobacillus* (24%), *Escherichia-Shigella* (6%), and *Enterococcus* (5%) (Figure 1B and Supplementary Table S1).

**Figure 2:**
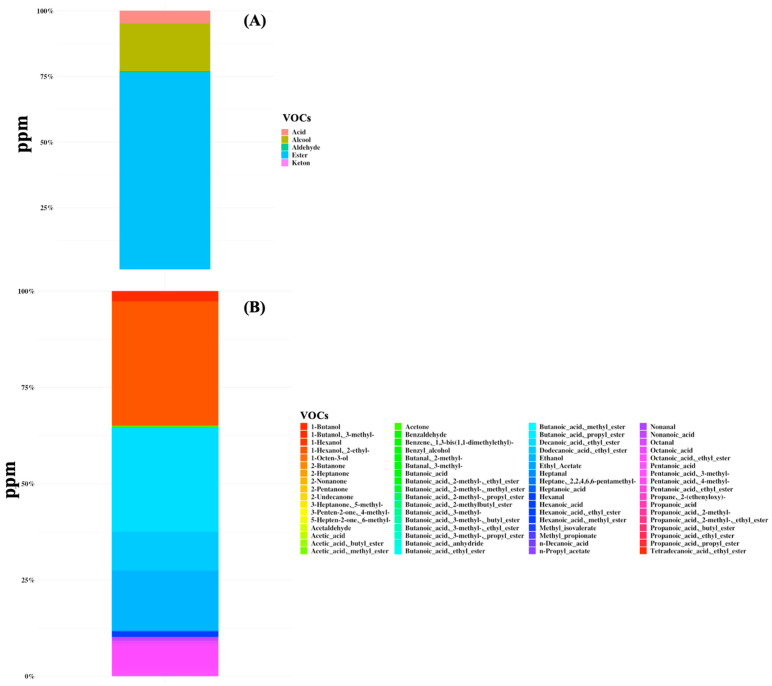
Volatile organic compounds (VOCs) were detected in gut samples of 44 SBS-CIF patients. The average concentration in ppm is displayed at the class level (**plot A**) or at the compound level (**plot B**).

A total of 68 volatile organic compounds were identified in all samples belonging to the class of organic acids, alcohols, aldehydes, esters, and ketones (Figure 2 and Supplementary Table S2). The total VOC content ranged from 0.5 to 3048 ppm and the metabolome was mainly characterized by the predominance of butanoic acid (mean amount of 45 ppm and the average percentage of presence was 45.8%) and hexanoic acid (40 ppm, 41.3%), followed by butanoic acid ethyl ester (10 ppm), octanoic acid (8 ppm), propanoic acid, 4-methyl-pentanoic acid (7 ppm), and acetic acid (6 ppm) (Figure 2). Moreover, high dispersion of data for both the microbiome and volatilome composition was observed and no specific signature was characteristic of any subgroup of patients.

### 3.3. Microbiome and Volatilome Characteristics According to Bowel Length and Underlying Diseases

By comparing patients with small bowel lengths of ≤100 cm and >100 cm, higher *Bacteroides* (*p* = 0.045) and *Lactobacilli* (*p* = 0.003) and lower *Pediococcus* (*p* = 0.030) and *Parvimonas* (*p* = 0.036) were found in patients with shorter lengths (≤100 cm). The frequency of *Saccharimonadaceae* (*p* = 0.010) and *Klebsiella* (*p* = 0.049) was significantly higher in patients with mesenteric ischemia.

Significantly higher concentrations of 1-butanol (*p* = 0.042) and lower concentrations of heptanal (*p* = 0.044) were found in patients with >100 cm bowel length.

No significant associations among microbiome and volatilome characteristics and bowel length and underlying diseases were found even if patients with small bowel length ≤ 100 cm displayed a trend (*p* = 0.058) in the accumulation of VOCs (more than 3000 ppm).

### 3.4. Microbiome and Volatilome Characteristics According to Functional Characteristics

The stomal output was directly associated with *Lactobacilli* (Rho = +0.38; *p* = 0.010) and inversely with *Erysipelatoclostridium* (Rho = −0.30; *p* = 0.047). Patients with a stomal output ≥ 1500 mL/day showed an increased frequency of *Lactobacillus* (*p* = 0.006) and *Prevotella* (*p* = 0.038), and a reduced frequency of *Erysipelatoclostridium (p* = 0.040) and *Intestinibacter* (*p* = 0.040). The direct association between *Lactobacillus* and high stomal output remained significant after adjusting for small bowel length (OR = 1.04; 95% CI 1.01–1.07, *p* = 0.023).

Patients with a stomal output ≥ 1500 mL/day showed increased concentrations in butanoic acid 2-methyl-propyl ester (*p* = 0.043). This association was not confirmed at multiple regression analysis.

### 3.5. Microbiome and Volatilome Characteristics According to Parenteral Supply

A significantly higher frequency of *Olsenella* (*p* = 0.042) and *Bacteroides* (*p* = 0.020) were found in patients with lower PN duration. No significant correlations between microbiota composition and energy infused were detected, while lower volume infused was associated with *Clostridium* (*p* = 0.046).

Regression analysis revealed no significant associations between parenteral supply and microbiome and volatilome characteristics.

### 3.6. Microbiome and Volatilome Characteristics According to Hyperphagia and Dietary Intakes

Seven participants had a FIR score > 1.5 and were considered to be hyperphagic. No significant association between hyperphagia and either the length of the small bowel, BMI, or the stomal output was found. Volume and energy infused did not differ between hyperphagic and non-hyperphagic participants.

**Figure 3:**
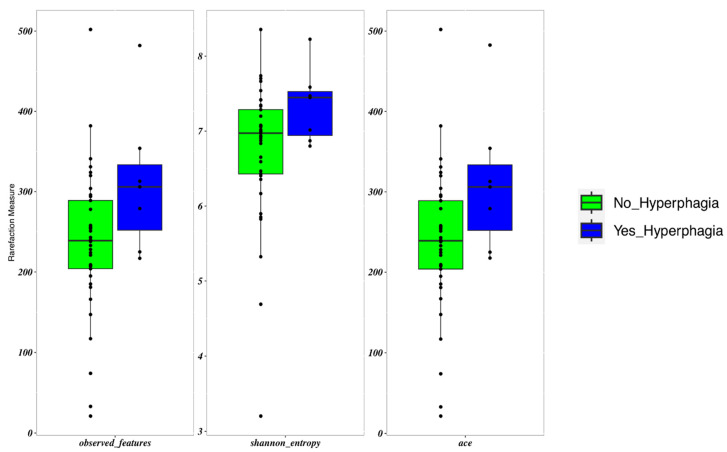
Boxplots to describe α-diversity measures of the microbiota of SBS-CIF patients with hyperphagia (FIR score > 1.5: blue bars, n = 7) compared to those without hyperphagia (FIR score ≤ 1.5: green bars, n = 37). Individual points and brackets represent the richness estimate and the theoretical standard error range, respectively.

Patients with hyperphagia showed an increased frequency of *Erysipelatoclostridium* (*p* = 0.017). These patients displayed a slightly higher α-diversity than those with lower FIR scores (*p* = 0.05) (Figure 3). FIR values were directly and significantly related to all measures of α-diversity. β-diversity did not differ between patients with and without hyperphagia.

No difference in α-diversity was evident between groups with different bowel lengths, underlying diseases, functional characteristics, parental supply, and dietary intakes. The same held true for β-diversity.

**Figure 4:**
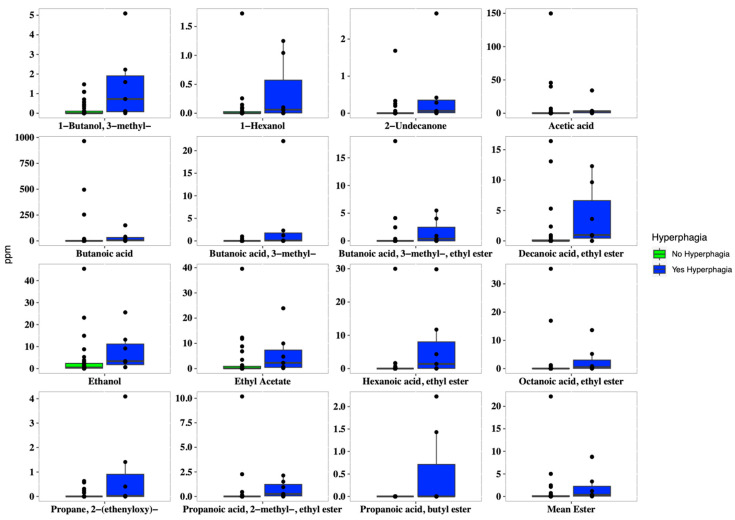
Boxplots of the concentrations of VOCs (expressed in ppm), which were significantly different (*p* < 0.05) in SBS-CIF patients with hyperphagia (FIR score > 1.5: blue bars; n = 7) compared to those without hyperphagia (FIR score ≤ 1.5: green bars, n = 37). Individual points and brackets represent the richness estimate and the theoretical standard error range, respectively.

In addition, hyperphagic patients showed a higher concentration of both several short and medium fatty esters, such as 1-butanol 3-methyl (*p* = 0.009), butanoic acid ethyl ester (*p* = 0.005), hexanoic acid ethyl ester (*p* = 0.004), methyl propionate (*p* = 0.049), acetic acid (*p* = 0.037), and butanoic acid (*p* = 0.038). Overall, patients with hyperphagia displayed higher concentrations of short-chain fatty esters, ethanol, and 2-undecanone (Figure 4).

**Figure 5:**
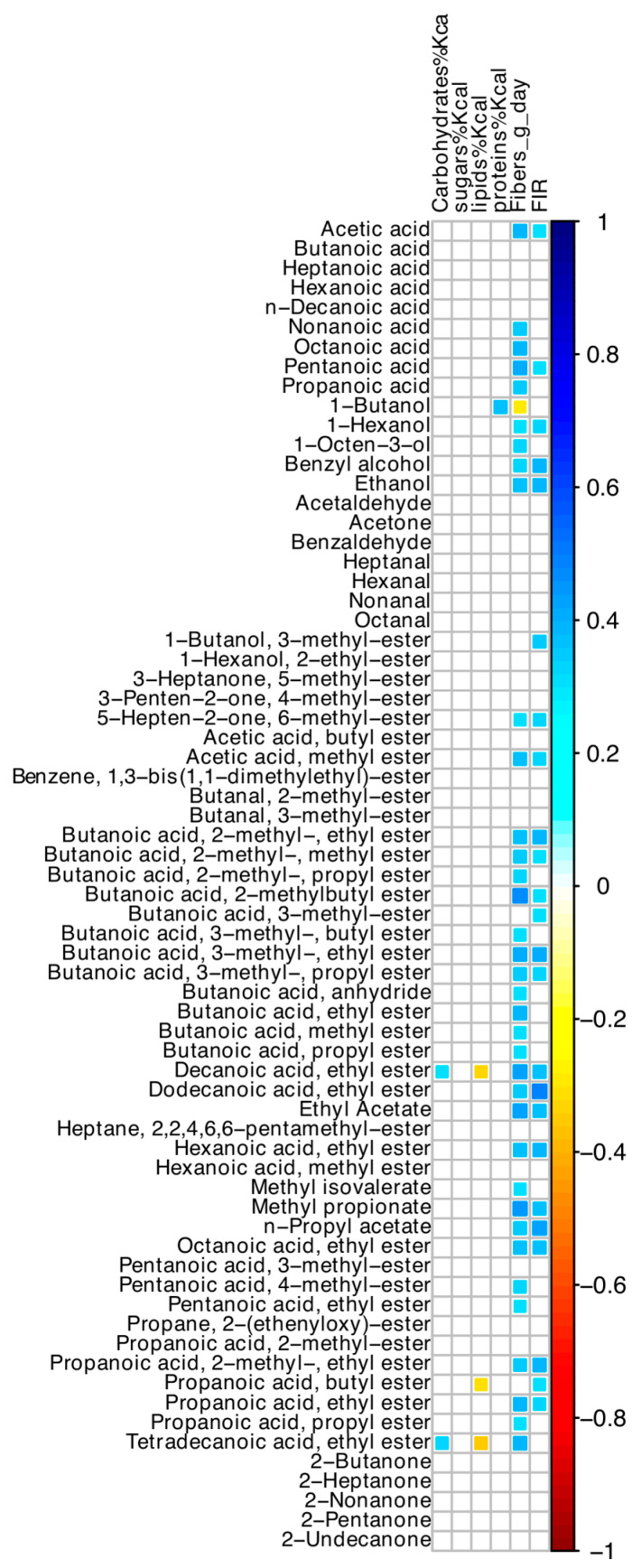
Spearman correlation between values of dietary intakes, FIR, and VOCs of SBS-CIF patients. The color of the scale bar denotes the nature of the correlation, with 1 indicating a perfectly positive correlation (dark blue) and −1 indicating a perfectly negative correlation (dark red). Only statistically significant correlations (*p* < 0.05) are shown.

Among nutrient intakes, oral intake of dietary fibers was directly correlated with most VOCs (Figure 5), but not with any ASV. Values of FIR and dietary fibers were significantly associated (r = +0.55; *p* < 0.001). Hyperphagia was associated with dietary fiber intake, after adjusting for small bowel length (OR = 1.35; 95% CI 1.01–1.81; *p* = 0.040).

### 3.7. Microbiome and Volatilome Characteristics According to hs-CRP Values

Hs-CRP values were inversely associated with *Bifidobacterium* (Rho = −0.42, *p* = 0.005) and directly associated with stomal output (Rho = +0.33, *p* = 0.048). This latter association was not confirmed in a regression model, after adjusting for small bowel length. On the other hand, hs-CRP ≥ 3 mg/L was inversely associated with *Bifidobacterium* in the same model (OR = 0.42; 95% CI 0.17–1.03, *p* = 0.049).

No association between hs-CRP values and volatilome features was found.

### 3.8. Correlations among Microbiome and Volatilome Characteristics

**Figure 6:**
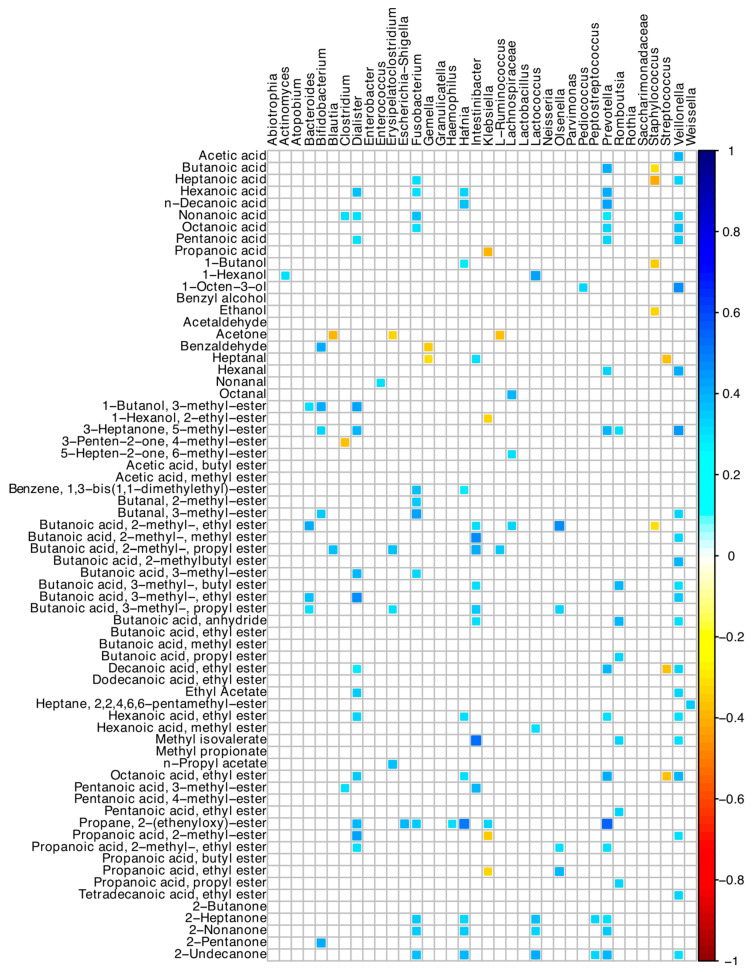
Spearman correlation between microbiota and VOCs of SBS-CIF patients. The color of the scale bar denotes the nature of the correlation, with 1 indicating a perfectly positive correlation (dark blue) and −1 indicating a perfectly negative correlation (dark red). Only statistically significant correlations (*p* < 0.05) are shown.

Many positive correlations between bacterial ASVs and the acid-volatile fractions were found (Figure 6). In detail, *Veillonella*, *Dialister*, *Intestinibacter*, and *Prevotella* ASVs were directly associated with many short- and medium-chain fatty acid compounds. *Prevotella* was associated with butanoic, hexanoic, decanoic, and nonanoic acids, and 2-heptanone and 2-nonanone, while *Bacteroides* with several esters of butanoic acid (Figure 6). *Staphylococcus*, *Streptococcus*, and *Klebsiella* showed negative correlations with a few of the detected VOCs.

## 4. Discussion

### 4.1. Microbiota Composition

The microbiota of the small bowel comprises facultative anaerobic species with predominant saccharolytic activity [8,30]. The removal of the colon and of a large part of the small bowel from intestinal transit causes oxygen enrichment, abnormal arrival of undigested nutrients, rapid transit time, disruption of enterohepatic circulation, and lumen acidification [8,31]. The ostomy exposes the gut microbiota to the skin and external environment [32]. Furthermore, in contrast to the stability in the microbiota composition of the colon (relatively low in nutrients), the microbiota of the small bowel (rich in nutrients) shows temporal dynamicity with differences between morning and afternoon profiles [32].

In our type 1 SBS-CIF patients, *Lactobacillaceae* and *Streptococcaceae* predominated with a higher relative abundance of Firmicutes (recently renamed Bacillota) at the expense of Bacteroidetes (recently renamed Bacteroidota), in line with the literature [8,9,10,12,13,14,15]. Both families contain facultative anaerobic, bile-acid resistant, carbohydrate-fermenting genera. The proximal part of the small bowel is an optimal ecosystem for *Lactobacilli* because of their bile resistance, low pH, and abundance of sugars [30]. In SBS patients, a higher amount of mono and disaccharides are available due to malabsorption, with increased production of lactic acid from sugar fermentation and increased acidity of the lumen, further favoring *Lactobacilli* growth [30].

At the genus level, *Streptococcus* was the predominant ASV in our patients, in line with the two other studies performed in type-1 SBS, showing an enrichment in *Streptococcus* [13,14]. This is intriguing since small bowel *Streptococci* are reported to be enriched with genes for energy generation and carbohydrate metabolism, thus considerably contributing to the primary digestion of foods [32]. Thus, it is conceivable that it is an adaptive mechanism to maximize food assimilation.

We found a few associations between microbiota composition and clinical characteristics of the patients. The most relevant was the greater frequency of *Lactobacilli* in the presence of greater stomal outputs. *Lactobacilli* have been correlated with a shorter duration for PN [10], but on the other hand, their excess has been shown to be deleterious by preventing the implantation of other beneficial bacteria [8], favoring SBS complications [9,15,33], deconjugating bile acids and impairing absorption of lipophilic substances [30], and being associated with increased oxidative stress [13]. Thus, at present, the exact role of *Lactobacilli* in SBS patients is far from being clarified. In view of these results, caution is needed when administering probiotics or fermented foods containing these genera to type 1 SBS-CIF individuals until the role of *Lactobacilli* in these patients is definitively clarified.

Our data are difficult to compare with the literature owing to the low number (≤10) of patients with type 1 SBS-CIF in the other two studies available from the literature [13,14]. Furthermore, other studies carried out comparisons between SBS patients and healthy controls, between the different types of SBS, and between patients weaned or not from PN, but not within type 1 SBS-CIF patients [13,14].

A small percentage of our type 1 SBS-CIF participants showed hyperphagia (16%). Hyperphagia has been reported to range between 43% and 83% in adult SBS patients [25,34,35]. The definition of hyperphagia is not homogeneous among studies; in this study, we used the more widely used definition, which refers to a food intake exceeding 1.5 times the REE [25,34,35]. We cannot exclude that the self-reported intakes of our patients were unreliable and led to under-reporting, thus justifying our low prevalence of hyperphagic patients. However, energy intakes were assessed by the same trained dietician, and dietary recommendations were standardized for all participants in accordance with guidelines. Previous studies included both patients with type 2 and 3 SBS and patients weaned from PN [25,34,35]. When disentangling individuals with a colon in continuity from type 1 SBS patients, a significantly lower percentage of hyperphagic patients was found in the latter individuals [25]. Furthermore, a lower caloric intake has been reported in PN-dependent compared to PN-weaned patients [36], although not all authors agreed on this association [35]. Other studies on type 1 SBS failed to find associations between FIR and BMI and the length of the residual small intestine, in accordance with our findings [25]. Finally, the significant association between hyperphagia and SCFAs (see below) has biological plausibility and supports the reliability of our data on hyperphagia.

An overall reduction in bacterial diversity and richness has been consistently reported in SBS patients when compared to healthy controls [15]. We found a higher alpha diversity in hyperphagic patients and a significant direct correlation between FIR values and measures of α-diversity. In our type 1 SBS-CIF patients, hyperphagia was not related to specific microbiota genera, but rather to their VOCs.

### 4.2. Volatile Organic Compounds

We identified significant correlations between a few ASVs, poorly represented in these patients, in particular *Intestinibacter* and *Prevotella*, and SCFAs, while neither *Lactobacilli* nor *Streptococci* appeared to be related to any of the identified VOCs. Very few studies are available in the literature to compare our results. In a murine model of SBS, higher contents of isoflurane, hexane, and pentane were reported with respect to mice undergoing sham operation, with the latter two being VOC markers of oxidative stress [37]. Budinska et al. described significantly higher concentrations of hexanal and nonanal in SBS patients when compared with controls [13]. Moreover, the authors reported increased levels of saturated aldehydes, medium-chain fatty acids, and particularly in type 1 SBS patients, reduced concentrations of SCFAs [13]. We observed a predominance of ketones and esters in our type 1 SBS-CIF patients, and SCFAs represented the main class of VOCs in most of our patients. Due to the different analytical procedures to determine and quantify VOCs, a direct comparison between studies is difficult.

Higher concentrations of short-chain fatty esters, ethanol, and 2-undecanone were found in hyperphagic patients. SCFAs are involved both in appetite stimulation—via the activation of ghrelin-related signaling and the inhibition of insulin secretion—and in appetite inhibition by stimulating the release of inhibitory peptides, such as glucagon-like-peptide 1, peptide YY, and leptin [38]. A direct role of brain neurons implicated in hunger-satiety regulation or the activation of reward mechanisms may be implicated too [39]. Thus, a possible role of the increased concentrations of SCFAs on appetite could be hypothesized, even if, due to the cross-sectional nature of this study, a causal relationship cannot be demonstrated. Furthermore, in our patients, neither hyperphagia nor the greater concentrations of SCFAs are associated with better clinical outcomes. In SBS patients, hyperphagia is beneficial in reducing the need for PN [25]. However, type 1 patients could have reduced their food intake to lower the stomal output and avoid repeated emptying of their stomal bags, thus probably influencing the lack of correlations between FIR and stomal output.

Finally, direct correlations between oral dietary fiber intake and most VOCs were evident in our patients, in line with the literature [39]. The correlation we found between oral fiber intake and hyperphagia could be modulated by the increase in SCFAs determined by dietary fiber intake. Therefore, it might be hypothesized that patients’ hyperphagia is linked to increased synthesis of SCFAs as a result of increased fiber ingestion. Nonetheless, in our patients, no correlation was observed between fiber intake and the microbiota. This could be due to our low sample size and/or to the high heterogeneity of our patients’ microbiota, which did not allow us to identify associations. It is possible, nevertheless, that additional, as-yet-unmeasured microorganisms (such as fungi) contributed to the dietary fiber breakdown in these type 1 SBS-CIF patients. Clinical trials with a prospective design are needed to define the role of fiber on VOC concentrations and the appetite of these patients.

### 4.3. Limitations

A control group of healthy individuals was lacking. We have considered that comparing the microbiome of patients without a colon with that of healthy people with intact intestines may be of little relevance due to the obvious and well-known differences already described by other studies in the literature.

The small sample size and the cross-sectional nature of this study were limitations that should be recognized. Indeed, SBS is a rare disease, and at present, this is the largest study on type 1 SBS-CIF patients. Furthermore, most available papers had a cross-sectional design, small sample size, and heterogeneous population characteristics, such as differences in site of intestinal resection and anastomoses, presence of ostomies, and underlying diagnoses, all of which are conditions that potentially impact local microbiota.

We did not analyze the metabolism of bile acids, which is highly impaired in SBS patients and may strongly disturb lipid metabolism [14,29,40]. Indeed, all of our patients have neither terminal ileum, where the reabsorption of 95% of bile salts occurs, nor the colon where the bile acids escaping the ileum reabsorption are metabolized. Thus, plausibly, all the patients were exposed to a similar environment high in biliary acids [40], which may have contributed to the selection of the bile acid-resistant microbiota we detected.

A great heterogeneity in clinical characteristics was evident among our patients, and this heterogeneity was reflected in their microbiome characteristics too. Indeed, we enrolled patients under strict inclusion criteria, such as neither recent use of drugs or supplements nor acute diseases or conditions potentially impacting microbiota.

## 5. Conclusions

The present research work represents the largest study assessing the microbiome characteristics of adult type 1 SBS-CIF patients. Even if a high heterogeneity was found within participants, a few common features were evident. A greater frequency of *Lactobacilli* was associated with increased stomal outputs, while higher fiber intake and concentrations of short-chain fatty esters were associated with hyperphagia. It is therefore possible that the microbiome plays a role in type 1 SBS-CIF patients. Our results might have implications for clinical practice but should be confirmed by other studies on a larger number of patients.

## Figures and Tables

**Table 1:** Clinical characteristics of the enrolled patients.

Variable	Sub-Classification	Number, Mean or Median Values
Number		44
Age (years)		64.2 ± 14.7
Females/males		25/19
Weight (kg)		63.2 ± 18.1
BMI (kg/m^2^)		23.2 ± 5.3
Underlying disease		
	Inflammatory bowel diseases	20 (45.5)
	Surgical complications	16 (36.4)
	Mesenteric ischemia	7 (15.9)
	Fibro-adhesive peritonitis	1 (2.3)
Small bowel length (cm)		124.2 ± 76.7
	≤100 cm	20 (45.5)
	>100 cm	24 (54.5)
Stomal output (mL/day)		1600; 1200
	≥1500 mL/day	25 (56.8)
Year of PN start		
	≤6 months	10 (22.7)
	6–24 months	5 (11.4)
	≥24 months	29 (65.9)
Total volume infused (mL/day)		1635.5 ± 896.4
	≥1500 mL/day	22 (50)
Fluid, oral intake (mL/day)		1458.0 ± 692.2
Energy, parenteral supply (kcal/day)		729.6 ± 435.1
Energy, parenteral supply/kg body weight (kcal/kg)/day		13.8 ± 7.1
	≥15 (kcal/kg) day	19 (43.2)
Energy, oral intake (kcal/day)		1439.2 ± 518.0
Food Intake Ratio (FIR score)		1.09 ± 0.39
	FIR score > 1.5	7 (15.9)
Serum hs-CRP (mg/L)		5.1; 5.8
	Hs-CRP ≥ 3 mg/L	29 (65.9)

Data are presented as mean ± SD, number (%), median, and interquartile range.

## Data Availability

Data presented in the study are available in the Supplementary Materials. Further inquiries can be directed to the Corresponding author.

## Supplementary Materials

The following supporting information can be downloaded at https://www.mdpi.com/article/10.3390/nu16142282/s1, Figure S1: Flowchart of the study population; Table S1: Microbiota composition at lowest taxonomic resolution of SBS-CIF patients; Table S2: Individual Volatile Organic Compounds (VOCs) express as ppm in stool samples of each SBS-CIF patient detected by using headspace (HS) solid-phase microextraction (SPME) coupled by gas chromatography-mass spectrometry (GC/MS).
