# Supplementary material for: Does Microbiome Matter in Chronic Intestinal Failure Due to Type 1 Short Bowel Syndrome in Adults?
Source: Nutrients. 2024 Jul 16;16(14):2282. doi: 10.3390/nu16142282 (PMC11280028; doi:10.3390/nu16142282)
Supplement: Supplementary file 1 [file nutrients-16-02282-s001.zip › nutrients-3073750-supplementary.pdf]

Supplementary Figure S1. Flowchart of the study population

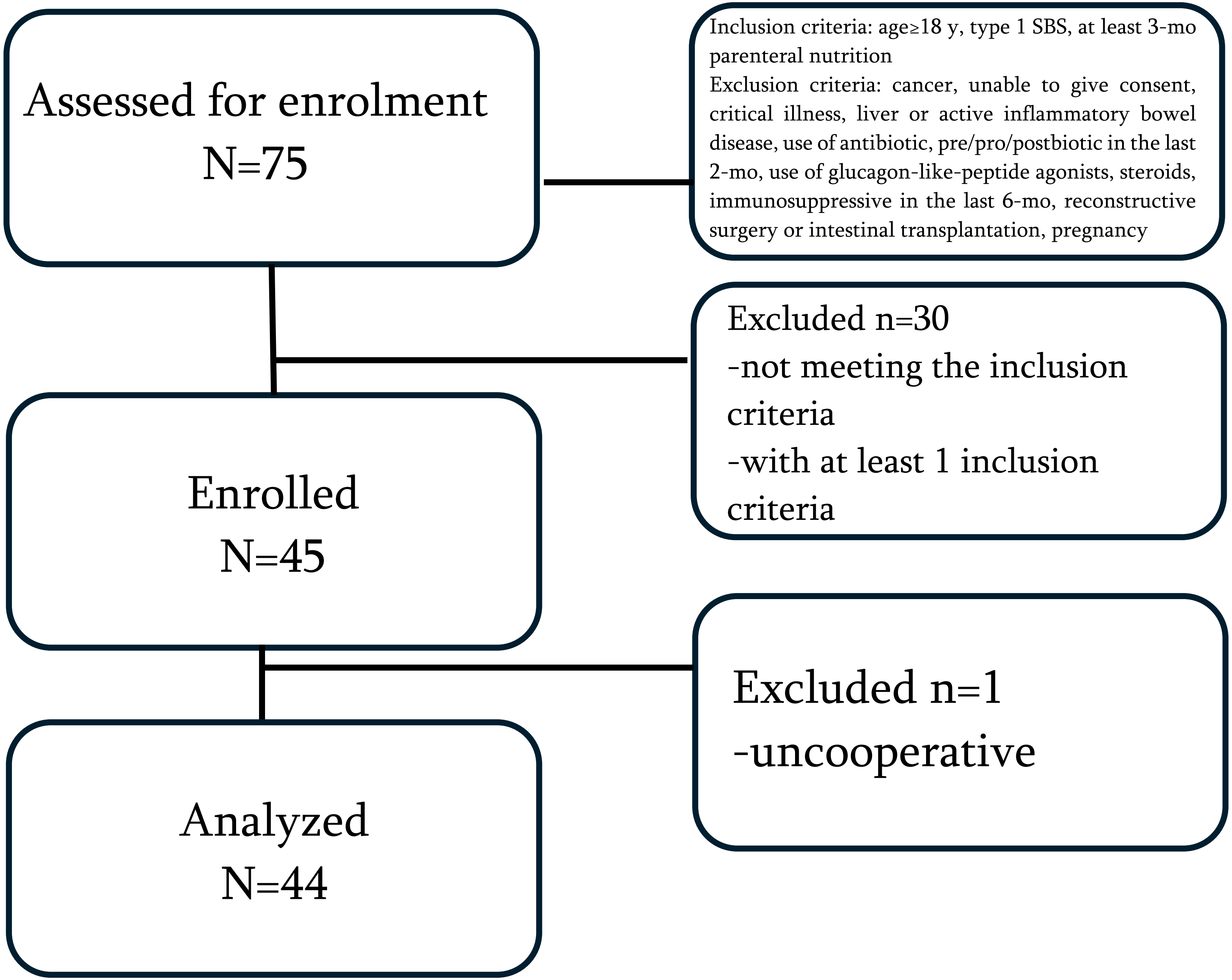

Supplementary Table S1. Microbiota composition at lowest taxonomic resolution of SBS-CIF patients.

Genus	1	2	3	4	5	6	7	8	9	10	11	12	13	14	15	16	17	18	19	20	21	22	23	24	25	26	27	28	29	30	31	32	33	34	35	36	37	38	39	40	41	42	43	44
Abiotrophia	0.00	0.00	0.03	0.00	0.10	0.00	0.20	0.00	0.00	0.00	0.00	0.00	0.00	0.00	0.00	0.00	0.00	0.00	0.00	0.00	0.00	0.00	0.00	0.00	0.00	0.00	0.00	12.0	0.00	0.00	0.00	0.00	0.00	0.00	0.10	0.00	2.14	0.00	2.02	0.00	0.00	0.00	0.00	0.00
Actinomyces	0.00	0.00	0.00	0.52	1.27	0.07	0.10	0.00	0.00	1.36	0.14	0.00	0.00	0.00	0.00	0.30	2.12	0.00	0.45	0.00	0.78	0.00	0.00	0.00	0.00	0.20	1.31	5.03	0.01	0.42	0.00	0.00	1.39	0.00	0.00	1.23	0.00	0.00	0.00	0.00	0.00	0.00	0.00	0.00
Atopobium	0.00	0.00	0.21	0.34	0.13	0.00	0.00	0.12	0.00	0.00	0.00	0.00	0.00	0.00	0.00	0.18	0.33	0.00	0.49	0.00	0.00	0.00	0.00	0.00	0.00	0.20	0.42	1.43	0.00	0.16	0.00	0.00	0.00	0.00	0.03	0.19	0.00	0.00	0.00	0.45	0.41	0.00	0.58	0.00
Bacteroides	0.00	0.00	0.00	0.00	0.00	0.00	0.00	0.00	0.00	0.00	0.00	0.01	0.26	2.53	0.00	0.00	0.00	0.00	0.00	0.00	0.00	0.00	0.00	0.00	0.00	0.00	0.00	0.00	0.00	0.00	0.00	0.06	0.00	1.41	0.00	0.00	0.00	0.00	0.00	0.00	0.00	16.4	0.00	0.00
Bifidobacterium	0.00	0.03	0.00	0.00	0.67	0.17	0.00	1.71	0.00	0.00	0.94	0.00	0.28	0.90	0.02	1.52	0.05	0.57	0.00	0.00	0.76	1.69	0.00	3.30	0.10	0.00	0.00	1.47	3.94	1.01	0.00	0.09	0.00	1.20	0.74	0.22	0.00	1.59	0.00	0.10	0.42	26.3	0.00	0.00
Blautia	0.00	0.00	0.00	0.00	0.00	0.00	0.00	0.00	0.00	4.62	0.00	0.00	26.5	0.00	0.00	0.00	0.00	0.00	0.00	0.00	0.00	0.00	0.00	6.25	0.00	0.00	0.00	0.00	0.00	0.00	0.00	0.00	0.00	0.00	0.00	0.00	0.00	0.00	0.00	0.00	0.00	0.00	0.00	0.00
Clostridium	1.19	24.6	0.00	1.13	0.08	27.6	0.57	0.00	56.7	0.15	3.43	0.10	0.06	0.00	0.17	1.03	0.00	0.00	0.46	0.15	0.01	0.01	0.00	7.31	0.00	0.00	0.00	0.00	0.04	0.42	0.00	0.00	0.26	0.00	37.6	1.20	0.00	0.00	0.00	0.00	0.00	0.21	0.01	11.1
Dialister	0.00	0.00	0.00	0.00	0.16	0.00	0.00	0.01	0.00	0.00	0.33	0.00	0.00	0.62	0.00	0.00	0.00	0.00	0.00	0.00	0.00	0.00	0.00	0.00	0.00	0.00	0.00	0.00	0.00	0.00	0.00	0.00	0.00	2.36	0.00	0.00	0.00	0.00	0.00	0.00	0.00	0.00	0.00	0.00
Enterobacter	0.00	0.00	0.00	0.00	0.00	0.30	0.80	2.55	0.00	0.00	0.31	0.00	0.00	0.00	0.00	0.00	0.00	0.00	0.00	0.00	0.00	0.00	0.00	0.00	0.00	0.00	0.57	0.00	0.00	0.00	0.00	0.00	0.00	0.00	0.00	0.00	8.16	0.00	0.00	0.00	0.00	0.00	0.00	11.0
Enterococcus	0.06	0.11	0.19	0.23	0.12	0.15	17.2	1.03	0.03	0.00	11.2	0.13	0.01	0.00	0.03	0.48	5.89	0.36	36.1	0.01	3.96	0.65	0.00	0.00	0.00	0.00	3.17	0.00	0.00	0.13	0.89	0.87	65.6	0.00	0.47	9.30	0.00	0.00	0.00	0.49	0.00	0.49	2.41	45.3
Erysipelatoclostridium	0.00	0.24	0.00	0.00	0.00	0.00	0.00	0.00	0.00	0.52	0.00	0.00	0.98	0.00	0.00	0.00	0.00	0.00	0.00	0.00	0.00	0.00	0.00	0.00	0.00	0.00	0.00	0.00	0.00	0.00	0.00	0.00	0.00	0.00	0.00	0.00	0.00	0.00	0.00	0.00	0.00	0.00	0.00	0.00
Escherichia-Shigella	6.38	1.13	1.40	14.1	1.85	6.91	53.7	0.04	39.7	17.4	2.12	0.38	0.13	33.2	17.0	0.00	0.03	34.8	0.35	0.85	1.54	0.00	0.19	3.67	0.20	0.01	0.47	0.02	11.9	0.45	3.67	0.35	0.40	0.57	0.56	0.16	0.00	0.96	0.11	0.39	0.00	8.89	25.5	0.44
Fusobacterium	0.02	0.00	0.39	0.22	6.35	0.00	0.00	0.47	0.00	0.00	4.34	0.00	0.00	0.13	0.00	0.14	0.00	0.00	0.00	0.00	0.00	0.00	0.00	0.00	0.00	0.11	0.00	0.00	0.00	0.20	0.00	0.00	0.08	0.00	0.00	0.25	0.00	0.00	0.00	0.00	0.00	0.32	3.07	0.00
Gemella	0.03	0.00	0.40	0.48	3.48	0.52	0.07	0.00	0.00	0.00	0.58	0.00	0.00	0.04	0.00	0.17	4.37	0.00	1.20	0.04	0.56	3.54	0.00	0.00	0.09	0.14	0.00	0.00	0.00	0.36	0.00	0.00	0.60	0.00	0.00	1.04	19.9	0.00	13.3	1.27	0.00	0.00	1.42	0.00
Granulicatella	0.00	0.03	0.25	1.33	1.29	0.33	0.10	0.00	0.00	0.16	0.00	0.07	0.14	0.00	0.00	0.39	1.89	0.00	1.05	0.17	0.37	0.21	0.00	0.00	0.00	0.11	1.17	1.20	0.07	0.19	1.94	0.00	0.20	0.00	0.54	1.23	5.18	0.00	10.3	4.04	0.00	0.00	1.13	0.46
Haemophilus	0.00	0.00	0.19	0.27	1.91	0.00	0.02	0.00	0.00	0.00	0.13	0.00	0.00	0.00	0.00	0.00	0.00	0.00	0.01	0.19	0.00	0.00	0.00	0.00	0.00	0.00	0.00	0.00	0.00	0.10	0.00	0.00	0.00	0.00	2.60	0.00	1.62	0.00	0.00	0.00	0.00	0.00	0.00	0.00
Hafnia	0.00	0.00	21.3	0.00	0.00	0.00	0.00	0.00	0.00	0.00	0.90	0.00	0.00	0.00	0.00	0.00	0.00	0.00	0.00	0.00	0.00	0.00	0.00	0.00	0.00	0.00	0.00	0.00	0.00	0.00	0.00	0.00	0.00	0.00	0.00	0.00	0.00	0.00	0.00	0.00	0.00	0.00	0.00	0.00
Intestinibacter	0.00	0.00	0.00	0.00	0.00	0.00	0.00	0.00	0.00	2.63	0.00	0.00	0.00	0.00	0.00	24.5	0.00	0.00	0.00	0.00	0.00	0.00	0.00	0.00	0.00	0.00	0.00	0.00	0.00	0.00	0.00	0.00	0.00	0.00	10.1	0.00	0.00	0.00	0.00	0.00	0.00	0.00	0.00	0.00

Klebsiella	5.62	19.99	0.39	18.4	2.72	1.42	13.0	0.00	0.00	0.26	0.00	0.02	0.00	0.00	0.26	1.77	0.00	0.03	13.3	7.16	0.33	0.00	0.00	0.00	0.00	0.00	3.01	0.00	0.00	0.00	0.00	0.84	0.00	0.00	0.00	0.00	0.00	0.00	0.00	1.67	0.00	0.00	0.01	0.00
L-Ruminococcus	0.00	0.00	0.00	0.00	0.00	0.00	0.00	0.00	0.00	9.79	0.00	0.00	3.58	0.00	0.00	0.00	0.00	0.00	0.00	0.00	0.00	0.00	0.00	64.0	0.00	0.00	0.00	0.00	0.00	0.00	0.00	0.00	0.00	0.00	0.00	0.00	0.00	0.00	0.00	0.00	0.00	0.00	0.00	0.00
Lachnospiraceae	0.00	0.00	0.00	0.00	0.00	0.00	0.00	0.00	0.07	3.30	0.00	0.00	12.7	0.00	0.00	0.00	0.08	0.00	0.00	0.00	0.00	0.00	0.00	0.00	0.00	0.00	0.00	0.09	0.00	0.17	0.00	0.00	0.00	0.00	0.00	0.00	0.00	0.24	0.00	0.00	0.00	0.00	0.00	0.00
Lactobacillus	72.7	47.4	30.5	1.67	5.26	19.1	0.95	22.3	0.01	1.21	15.8	77.4	0.93	50.5	14.4	1.90	3.18	39.0	0.16	8.67	15.9	22.1	74.9	0.15	8.09	63.8	28.2	0.39	64.6	52.9	8.51	88.7	19.9	79.4	0.11	1.28	0.00	41.4	0.77	2.56	41.9	38.0	5.02	1.99
Lactococcus	0.00	0.00	0.41	0.00	0.00	1.07	0.47	0.00	0.00	0.10	14.6	0.00	0.00	0.00	0.00	0.00	4.04	6.40	0.00	0.00	5.98	0.00	0.00	0.00	0.00	0.00	0.00	0.00	0.00	0.00	0.00	0.67	0.47	0.00	0.00	44.9	0.00	0.00	0.00	0.00	0.00	0.00	0.00	0.15
Neisseria	0.00	0.00	0.08	0.00	2.56	0.00	0.00	0.00	0.00	0.00	0.00	0.00	0.00	0.00	0.00	0.00	0.00	0.00	0.00	0.00	0.00	0.00	0.00	0.00	0.00	0.00	0.00	0.00	0.00	0.00	0.00	0.00	0.00	0.00	0.00	0.00	7.38	0.00	0.00	0.18	0.00	0.00	0.00	0.00
Olsenella	0.00	0.00	0.00	0.00	0.00	0.00	0.00	0.00	0.00	0.00	0.00	0.00	0.00	0.03	0.00	0.00	0.00	0.00	0.00	0.00	0.00	0.00	0.00	0.00	0.00	0.00	0.00	3.29	0.00	0.00	0.00	0.00	0.00	0.62	0.00	0.00	0.00	0.00	0.00	0.00	0.00	0.00	0.00	0.00
Parvimonas	0.00	0.00	0.00	0.00	0.60	0.00	0.00	0.22	0.00	0.00	0.00	0.00	0.01	0.00	0.00	0.00	0.00	0.00	0.00	0.00	0.00	0.00	0.00	0.00	0.00	0.00	0.16	0.73	0.00	0.30	0.00	0.00	0.05	0.00	0.00	0.41	0.00	0.00	2.29	0.10	0.00	0.00	0.05	0.00
Pediococcus	0.00	0.00	0.00	0.00	20.6	0.00	0.00	0.00	0.00	0.00	0.00	0.00	0.00	0.00	0.00	0.67	0.00	0.00	0.00	0.00	0.00	0.00	0.00	0.00	0.00	0.00	1.43	0.00	0.00	0.00	0.56	0.00	0.00	0.00	0.00	2.21	0.00	0.00	0.00	0.00	0.00	0.00	0.00	0.00
Peptostreptococcus	0.00	0.00	0.04	0.03	0.81	0.00	0.00	0.00	0.00	0.00	0.10	0.00	0.00	0.00	0.00	0.00	0.00	0.02	0.06	0.00	0.00	0.00	0.00	0.00	0.00	0.00	0.14	2.63	0.00	0.20	0.00	0.00	0.12	0.00	0.00	0.42	0.00	0.00	0.00	0.00	0.00	0.00	0.10	0.00
Prevotella	0.00	0.00	0.09	0.15	1.54	0.00	0.00	0.00	0.00	0.00	0.24	0.00	0.00	0.00	0.00	0.00	0.00	0.00	0.00	0.00	0.00	0.00	0.00	0.00	0.00	0.00	0.00	0.00	0.00	0.00	0.00	0.00	0.00	7.03	0.00	0.00	0.00	0.00	0.00	0.00	0.00	0.00	0.00	0.00
Romboutsia	0.00	0.00	0.00	0.00	0.00	0.00	6.36	0.00	0.00	0.00	0.00	0.00	0.00	0.00	0.00	0.00	0.00	0.00	0.00	0.00	0.00	0.00	0.00	0.00	0.00	0.00	0.00	0.00	0.00	0.00	0.00	0.00	0.00	0.00	1.24	0.00	0.00	0.00	0.00	0.00	0.00	0.00	0.00	0.00
Rothia	002	0.00	0.07	0.50	0.89	0.79	0.12	0.78	0.00	0.53	0.00	0.27	0.01	0.00	0.00	1.57	3.81	0.00	3.74	0.25	9.64	9.35	0.00	0.00	3.15	1.43	0.28	0.11	0.40	0.78	3.65	0.00	0.32	0.00	0.22	5.49	0.00	0.02	1.91	10.7	3.80	0.00	1.86	5.11
Saccharimonadaceae	0.00	0.00	0.07	1.82	0.37	0.00	0.00	0.00	0.00	0.04	0.00	0.00	0.00	0.00	0.01	0.00	1.07	0.00	0.07	0.00	0.00	0.00	0.00	0.00	0.00	0.00	0.00	0.13	0.00	0.00	0.00	0.00	0.08	0.00	0.00	0.00	0.00	0.00	0.00	0.00	0.00	0.00	0.31	0.00
Staphylococcus	0.01	0.00	0.00	0.00	0.20	0.10	0.08	0.07	0.00	0.00	0.00	0.00	0.00	0.00	0.00	0.06	0.30	0.00	0.00	0.00	0.10	1.98	0.00	0.00	0.00	0.00	0.61	0.00	0.00	0.00	0.00	0.00	0.00	0.00	0.00	0.00	4.21	0.00	0.00	0.00	0.00	0.00	0.00	0.00
Streptococcus	10.9	3.66	35.8	41.1	37.0	38.7	5.54	68.8	0.86	52.3	36.9	13.0	12.2	11.4	67.5	64.5	69.3	18.8	40.5	80.3	59.9	59.2	24.8	14.1	87.4	32.5	54.5	65.0	18.6	38.7	80.6	6.85	8.59	0.03	31.7	26.9	41.9	46.2	69.3	76.9	53.3	2.32	53.6	23.8
Veillonella	2.85	2.09	6.92	16.0	7.73	0.20	0.55	0.91	0.42	1.13	7.36	1.04	0.00	0.00	0.30	0.50	0.79	0.00	1.24	2.15	0.00	0.73	0.00	0.00	0.39	1.32	4.57	0.19	0.53	3.15	0.00	0.90	0.71	3.81	13.6	3.15	0.00	9.56	0.00	0.39	0.00	6.88	3.95	0.00
Weissella	0.00	0.00	0.00	0.00	0.00	0.00	0.00	0.00	0.00	0.00	0.00	7.60	0.00	0.00	0.00	0.00	2.03	0.00	0.00	0.00	0.00	0.00	0.00	0.00	0.31	0.00	0.00	0.00	0.00	0.00	0.00	0.00	0.00	0.00	0.00	0.00	0.00	0.00	0.00	0.00	0.00	0.00	0.00	0.50

Amplicon sequence variants (ASVs) filtered at 0.5% are displayed if present in at least two samples

Supplementary Table S2. Individual Volatile Organic Compounds (VOCs) express as ppm in stool samples of each SBS-CIF patient detected by using headspace (HS) solid-phase microextraction (SPME) coupled by gas chromatography-mass spectrometry (GC/MS).

VOCs (ppm)	1	2	3	4	5	6	7	8	9	10	11	12	13	14	15	16	17	18	19	20	21	22	23	24	25	26	27	28	29	30	31	32	33	34	35	36	37	38	39	40	41	42	43	44
Acetic acid	0.00	2.94	3.55	0.17	0.50	0.00	0.00	0.00	0.00	0.00	0.00	0.56	0.00	1.18	1.48	0.00	0.00	0.00	0.00	0.00	1.14	0.00	0.00	0.00	3.30	0.00	0.00	0.00	0.00	6.86	0.00	0.00	45.7	40.1	149	3.38	0.00	34.2	0.00	0.00	0.00	0.00	1.57	0.10
Butanoic acid	0.00	4.03	25.4	3.19	1.96	0.00	0.00	0.00	0.06	0.01	11.2	0.29	0.01	6.30	6.99	0.00	0.00	0.00	0.00	0.00	5.51	0.00	0.00	0.00	19.5	0.32	0.00	0.00	0.00	0.00	0.00	0.10	495	254	964	40.7	0.03	150	0.02	0.09	0.00	0.00	0.00	0.62
Heptanoic acid	0.00	0.12	0.54	0.05	0.00	0.00	0.00	0.00	0.00	0.00	0.12	0.00	0.02	0.24	0.10	0.00	0.00	0.00	0.00	0.00	0.00	0.00	0.00	0.00	0.01	0.00	0.00	0.00	0.00	2.20	0.00	0.00	18.7	0.21	0.85	0.14	0.00	2.84	0.00	0.00	0.00	0.00	0.85	1.42
Hexanoic acid	0.01	1.50	30.7	3.32	1.48	0.06	0.00	0.28	0.02	0.01	10.3	0.00	0.01	9.30	5.37	0.00	0.01	0.00	0.00	0.00	9.55	0.00	0.00	0.00	0.11	0.00	0.00	0.00	0.00	0.00	0.00	0.00	1358	6.53	24.8	3.53	0.00	325	0.06	0.07	0.00	0.00	2.04	2.95
n-Decanoic acid	0.00	0.04	0.88	0.19	0.00	0.00	0.00	0.00	0.00	0.00	0.67	0.00	0.00	0.16	0.18	0.00	0.00	0.00	0.00	0.00	2.13	0.00	0.00	0.00	0.00	0.00	0.00	0.00	0.00	0.00	0.00	0.00	11.9	0.91	1.49	0.00	0.00	0.37	0.00	0.01	0.00	0.00	0.00	0.00
Nonanoic acid	0.01	0.43	1.55	0.25	0.28	0.04	0.02	0.28	0.05	0.00	0.79	0.84	0.01	0.87	0.74	0.00	0.01	0.00	0.00	0.00	0.53	0.00	0.00	0.00	0.10	0.00	0.00	0.00	0.00	6.50	0.00	0.00	6.29	0.34	2.45	0.21	0.00	0.47	0.00	0.00	0.00	0.00	1.87	3.77
Octanoic acid	0.01	0.16	1.56	0.23	0.06	0.00	0.00	0.00	0.00	0.00	1.08	0.68	0.00	1.53	0.35	0.00	0.00	0.00	0.00	0.00	7.75	0.00	0.00	0.00	0.03	0.00	0.00	0.00	0.00	3.12	0.00	0.00	304	2.10	7.40	0.67	0.00	24.3	0.00	0.01	0.00	0.00	1.30	2.71
Pentanoic acid	0.01	0.16	0.91	0.00	0.10	0.00	0.00	0.00	0.01	0.00	0.38	1.04	0.00	0.11	0.29	0.00	0.00	0.00	0.00	0.00	0.72	0.00	0.00	0.00	0.07	0.00	0.00	0.00	0.00	5.14	0.00	0.00	15.2	9.25	35.2	0.00	0.00	4.71	0.00	0.00	0.00	0.00	0.00	0.00
Propanoic acid	0.00	0.00	0.00	0.00	0.00	0.00	0.00	0.00	0.00	0.00	0.37	0.00	0.00	0.00	0.00	0.00	0.00	0.00	0.00	0.00	0.00	0.00	0.00	0.00	0.45	0.00	0.00	0.00	0.00	0.00	0.00	0.00	9.17	42.8	162	3.20	0.00	104	0.00	0.00	0.00	0.00	0.00	0.11
1-Butanol	0.02	0.19	1.16	0.03	0.00	0.00	0.00	0.00	0.71	0.03	2.17	0.00	0.15	0.00	0.00	0.02	0.00	0.00	0.00	0.01	0.00	0.00	1.26	0.00	0.51	0.00	0.72	6.98	0.24	0.00	0.00	0.19	0.00	0.00	0.00	4.38	0.00	23.7	2.39	0.06	0.03	0.08	0.03	0.35
1-Hexanol	0.00	0.00	0.00	0.00	0.09	0.06	0.03	0.00	0.02	0.01	0.00	0.00	0.00	0.10	0.00	0.00	0.03	0.00	0.00	0.10	1.73	0.00	0.01	0.00	0.03	0.00	0.00	0.01	0.09	0.00	0.00	0.15	0.26	0.00	0.00	1.04	0.00	1.25	0.00	0.00	0.00	0.00	0.00	0.05
1-Octen-3-ol	0.00	0.00	0.00	0.00	0.15	0.00	0.00	0.00	0.05	0.02	0.00	0.00	0.00	0.00	0.00	0.00	0.00	0.00	0.00	0.01	0.00	0.00	0.00	0.01	0.03	0.04	0.10	0.00	0.00	0.18	0.00	0.00	3.47	0.22	0.78	0.27	0.00	0.97	0.00	0.00	0.00	0.07	0.00	0.00
Benzyl alcohol	0.00	0.00	1.15	0.00	0.00	0.63	0.01	0.00	0.00	0.13	0.00	0.00	0.86	3.72	12.8	2.71	0.05	0.15	0.01	0.01	0.00	0.01	9.29	0.42	0.09	13.0	9.38	0.00	0.15	53.2	0.11	0.00	0.00	5.61	21.1	1.95	0.00	20.3	0.00	0.00	0.00	0.00	0.00	24.3
Ethanol	0.09	2.94	3.38	0.30	1.40	0.63	1.11	2.03	1.19	0.63	2.21	45.4	0.30	13.2	8.77	0.19	0.14	0.14	0.01	0.59	3.21	0.01	0.31	0.66	2.39	0.46	0.24	1.15	0.43	2.88	0.01	0.24	23.1	3.88	14.9	9.16	0.01	25.6	0.38	0.07	0.04	0.29	5.29	3.43
Acetaldehyde	0.00	0.00	0.09	0.00	0.29	0.00	0.03	0.20	0.02	0.00	0.00	0.52	0.02	0.13	0.33	0.01	0.00	0.02	0.00	0.01	0.61	0.00	0.00	0.00	0.01	0.14	0.00	0.01	0.12	0.00	0.00	0.00	0.00	0.00	0.00	0.00	0.00	2.13	0.00	0.00	0.00	0.10	0.03	0.01
Acetone	0.00	0.03	0.22	0.13	0.38	0.02	0.03	0.24	0.08	0.01	0.38	0.85	0.00	0.02	1.74	0.01	0.07	0.00	0.04	0.01	1.52	0.01	0.03	0.02	0.02	0.60	0.02	0.14	0.44	1.37	0.13	0.09	1.46	0.14	0.53	0.36	0.09	2.30	0.03	0.04	0.03	0.02	0.04	0.88

Benzaldehyde	0.00	0.00	0.00	0.00	0.23	0.02	0.12	0.00	0.14	0.04	0.00	0.00	0.55	0.13	0.51	0.29	0.00	0.30	0.00	0.05	0.75	0.00	0.08	0.11	0.05	0.00	0.65	0.65	0.43	0.65	0.00	0.05	1.28	0.63	2.61	0.25	0.00	2.44	0.00	0.00	0.00	0.18	0.00	0.06
Heptanal	0.00	0.00	0.00	0.00	0.00	0.00	0.00	0.00	0.11	0.01	0.00	0.00	0.00	0.00	0.00	0.00	0.00	0.00	0.00	0.00	0.00	0.00	0.00	0.00	0.00	0.00	0.03	0.00	0.03	0.00	0.00	0.00	8.15	0.36	1.49	0.00	0.00	0.00	0.00	0.00	0.00	0.09	0.00	0.00
Hexanal	0.00	0.57	0.86	0.13	1.62	0.01	0.00	0.00	0.18	0.00	0.11	2.64	0.00	0.08	1.16	0.01	0.00	0.00	0.00	0.01	35.6	0.00	0.00	0.05	0.01	0.06	0.27	0.04	0.03	1.23	0.01	0.09	60.1	0.62	1.94	0.20	0.04	10.8	0.00	0.00	0.00	0.09	0.00	0.02
Nonanal	0.00	0.02	0.36	0.04	0.30	0.08	0.05	0.36	0.10	0.04	0.49	1.09	0.03	0.23	0.97	0.06	0.09	0.06	0.05	0.00	0.71	0.02	0.01	0.03	0.00	0.04	0.04	0.00	0.06	19.2	0.02	0.02	26.3	0.38	1.31	0.10	0.06	4.14	0.03	0.02	0.01	0.00	2.45	4.72
Octanal	0.00	0.00	0.00	0.00	0.00	0.00	0.01	0.00	0.03	0.05	0.00	0.00	0.02	0.00	0.00	0.02	0.00	0.00	0.00	0.01	0.06	0.00	0.00	0.00	0.06	0.00	0.03	0.04	0.03	4.04	0.00	0.00	8.98	0.40	1.35	0.00	0.00	1.51	0.00	0.00	0.00	0.00	1.09	1.86
1-Butanol, 3-methyl-	0.00	0.00	0.72	0.00	0.11	0.05	0.00	0.70	0.02	0.11	1.09	1.47	0.04	5.09	0.00	0.04	0.11	0.04	0.00	0.02	0.57	0.00	0.00	0.01	0.00	0.00	0.00	0.00	0.25	0.00	0.00	0.00	0.00	0.36	1.09	1.59	0.00	2.23	0.00	0.00	0.00	0.45	0.03	0.09
1-Hexanol, 2-ethyl-	0.18	0.02	0.00	0.04	0.12	0.25	0.04	0.00	0.03	0.07	0.83	0.00	0.08	7.52	0.91	0.31	0.14	0.00	0.05	0.18	0.00	0.06	0.33	0.12	0.20	0.24	0.09	0.56	0.16	3.53	0.37	0.31	0.00	0.20	0.44	0.00	0.06	0.17	2.65	0.05	0.27	0.33	1.52	1.90
3-Heptanone, 5-methyl-	0.00	0.00	0.00	0.00	0.00	0.00	0.00	0.00	0.00	0.00	0.29	0.00	0.00	0.00	0.00	0.00	0.00	0.00	0.00	0.00	0.00	0.00	0.00	0.00	0.00	0.00	0.00	0.00	0.00	0.00	0.00	0.00	0.00	0.36	1.60	0.00	0.00	1.79	0.00	0.00	0.00	0.00	0.00	0.00
3-Penten-2-one, 4-methyl-	0.00	0.00	0.00	0.00	0.00	0.00	0.00	0.00	0.00	0.00	0.00	0.00	0.00	0.00	0.00	0.00	0.00	0.00	0.00	0.00	0.43	0.00	0.05	0.00	0.00	0.00	0.03	0.61	0.00	0.00	0.00	0.00	0.00	0.00	0.00	0.00	0.00	2.48	0.14	0.00	0.00	0.00	0.00	0.00
5-Hepten-2-one, 6-methyl-	0.00	0.00	0.00	1.13	0.38	0.02	0.03	0.00	0.10	0.03	0.00	0.80	0.08	0.63	0.53	0.00	0.00	0.00	0.00	0.00	0.00	0.00	0.00	0.00	0.00	0.00	0.00	0.08	0.00	0.59	0.13	0.03	2.96	0.21	0.84	0.71	0.00	5.48	0.00	0.00	0.00	0.00	0.00	0.00
Acetic acid, butyl ester	0.00	0.00	0.00	0.00	0.00	0.00	0.00	0.00	0.00	0.00	0.00	0.00	0.00	0.00	0.00	0.00	0.00	0.00	0.00	0.00	0.00	0.00	0.01	0.00	0.14	0.00	0.00	0.00	0.00	0.00	0.00	0.00	0.00	0.57	1.87	2.50	0.00	0.34	0.00	0.00	0.00	0.00	0.35	0.00
Acetic acid, methyl ester	0.00	0.00	0.00	0.00	0.14	0.01	0.00	0.00	0.00	0.03	0.00	0.00	0.00	0.00	0.43	0.00	0.00	0.00	0.00	0.00	1.14	0.00	0.19	0.00	0.14	0.00	0.00	0.00	0.00	0.00	0.00	0.00	0.00	1.72	7.81	1.03	0.00	0.57	0.00	0.00	0.00	0.00	0.00	0.00
Benzene, 1,3-bis(1,1-dimethylethyl)-	0.00	0.00	1.10	0.47	0.00	0.00	0.40	0.71	0.08	0.05	0.50	0.00	0.02	0.21	0.00	0.09	0.00	0.00	0.15	0.08	1.20	0.06	0.00	0.00	0.00	0.00	0.00	0.00	0.56	0.92	0.00	0.00	3.50	0.00	0.00	0.00	0.00	3.76	0.22	0.07	0.09	0.45	0.18	0.21
Butanal, 2-methyl-	0.00	0.00	0.23	0.08	0.29	0.01	0.05	0.07	0.03	0.01	0.16	1.24	0.12	0.12	0.15	0.09	0.00	0.18	0.00	0.02	1.12	0.00	0.00	0.02	0.00	0.20	0.00	0.21	0.01	0.23	0.00	0.09	0.00	0.00	0.00	0.00	0.00	1.98	0.00	0.02	0.00	0.23	0.07	0.05
Butanal, 3-methyl-	0.00	0.02	0.53	0.31	0.70	0.02	0.09	0.17	0.08	0.01	0.47	3.21	0.23	0.33	0.34	0.10	0.01	0.21	0.00	0.03	3.37	0.00	0.00	0.04	0.00	0.58	0.00	0.34	0.27	0.48	0.01	0.20	1.00	0.11	0.37	0.16	0.00	9.98	0.01	0.04	0.00	0.74	0.15	0.03
Butanoic acid, 2-methyl-, ethyl ester	0.00	0.00	0.00	0.00	0.00	0.00	0.00	0.00	0.00	0.18	0.00	0.75	0.03	0.22	0.00	0.00	0.00	0.00	0.00	0.01	0.00	0.00	0.00	0.00	0.00	0.00	0.00	0.01	0.00	0.00	0.00	0.00	0.00	7.23	30.3	10.1	0.00	0.25	0.00	0.00	0.00	0.00	0.00	0.00
Butanoic acid, 2-methyl-, methyl ester	0.00	0.00	0.00	0.00	0.00	0.00	0.00	0.00	0.00	0.02	0.00	0.00	0.00	0.00	0.00	0.00	0.00	0.00	0.00	0.00	0.00	0.00	0.00	0.00	0.00	0.00	0.00	0.00	0.00	0.00	0.00	0.00	0.00	0.64	2.76	0.89	0.00	0.00	0.00	0.00	0.00	0.00	0.00	0.00

Butanoic acid, 2-methyl-, propyl ester	0.00	0.00	0.00	0.00	0.00	0.00	0.00	0.00	0.00	0.02	0.00	0.00	0.01	0.00	0.00	0.00	0.00	0.00	0.00	0.11	0.00	0.00	0.00	0.00	0.00	0.00	0.00	0.00	0.00	0.00	0.00	0.00	0.00	3.74	15.4	1.70	0.00	0.00	0.00	0.00	0.00	0.00	0.00	0.00
Butanoic acid, 2-methylbutyl ester	0.00	0.00	0.00	0.00	0.00	0.00	0.00	0.00	0.00	0.00	0.00	0.00	0.00	0.00	0.00	0.00	0.01	0.00	0.00	0.01	0.00	0.00	0.00	0.00	0.00	0.00	0.00	0.00	0.00	0.00	0.00	0.00	0.00	0.41	1.10	0.40	0.00	1.11	0.00	0.00	0.00	0.00	0.00	0.00
Butanoic acid, 3-methyl-	0.00	0.08	0.00	0.00	0.13	0.00	0.00	0.00	0.00	0.00	0.47	0.00	0.00	1.22	0.00	0.00	0.00	0.00	0.00	0.01	0.00	0.00	0.00	0.00	0.00	0.00	0.00	0.00	0.00	0.00	0.00	0.00	0.98	0.00	0.00	22.1	0.00	2.30	0.00	0.00	0.00	0.00	0.00	0.00
Butanoic acid, 3-methyl-, butyl ester	0.00	0.00	0.00	0.00	0.00	0.00	0.00	0.00	0.00	0.00	0.00	0.00	0.00	0.00	0.00	0.00	0.00	0.00	0.00	0.00	0.00	0.00	0.00	0.00	0.00	0.00	0.00	0.00	0.00	0.00	0.00	0.00	0.00	0.28	0.81	0.77	0.00	0.00	0.00	0.00	0.00	0.00	0.00	0.00
Butanoic acid, 3-methyl-, ethyl ester	0.00	0.00	0.00	0.00	0.17	0.00	0.00	0.00	0.00	0.38	0.36	2.44	0.06	4.03	0.00	0.00	0.00	0.00	0.00	0.11	0.00	0.00	0.00	0.00	0.00	0.00	0.00	0.00	0.00	0.00	0.00	0.00	0.00	4.12	18.0	5.48	0.00	0.91	0.00	0.00	0.00	0.00	0.00	0.00
Butanoic acid, 3-methyl-, propyl ester	0.00	0.00	0.00	0.00	0.00	0.00	0.00	0.00	0.00	0.13	0.00	0.00	0.02	0.11	0.00	0.00	0.00	0.00	0.00	0.33	0.00	0.00	0.00	0.00	0.04	0.00	0.00	0.00	0.00	0.00	0.00	0.00	0.00	5.79	23.3	1.42	0.00	0.00	0.00	0.00	0.00	0.00	0.00	0.00
Butanoic acid, anhydride	0.00	0.00	0.00	0.00	0.00	0.00	0.00	0.00	0.00	0.00	0.00	0.00	0.00	0.00	0.00	0.00	0.00	0.00	0.00	0.00	0.00	0.00	0.00	0.00	0.00	0.00	0.00	0.00	0.00	0.00	0.00	0.00	0.00	1.16	3.93	1.56	0.00	0.00	0.00	0.00	0.00	0.00	0.00	0.00
Butanoic acid, ethyl ester	0.00	1.83	1.37	0.00	0.00	0.00	0.00	0.00	0.00	0.00	0.00	0.00	0.00	2.07	0.00	0.00	0.00	0.00	0.00	0.03	0.00	0.00	0.00	0.00	23.6	0.00	0.00	0.00	0.00	0.00	0.00	0.00	6.82	41.3	120	255	0.01	6.21	0.00	0.02	0.00	0.00	0.00	0.00
Butanoic acid, methyl ester	0.00	0.00	0.00	0.00	0.00	0.00	0.00	0.00	0.00	0.00	0.00	0.00	0.00	0.00	0.00	0.00	0.00	0.00	0.00	0.00	0.47	0.00	0.00	0.00	0.99	0.00	0.00	0.00	0.00	0.00	0.00	0.00	0.00	0.40	8.02	10.3	0.00	0.00	0.00	0.00	0.00	0.00	0.00	0.00
Butanoic acid, propyl ester	0.00	0.00	0.00	0.00	0.00	0.00	0.00	0.00	0.00	0.00	0.00	0.00	0.00	0.00	0.00	0.00	0.00	0.00	0.00	0.00	0.00	0.00	0.00	0.00	1.80	0.00	0.00	0.00	0.00	0.00	0.00	0.00	0.00	12.6	50.9	2.78	0.00	0.00	0.00	0.00	0.00	0.00	0.00	0.00
Decanoic acid, ethyl ester	0.15	0.87	0.96	0.16	0.18	0.01	0.03	0.00	0.00	0.00	0.93	16.4	0.00	3.62	0.34	0.00	0.00	0.00	0.00	0.00	0.64	0.00	0.00	0.01	0.00	0.00	0.00	0.00	0.00	0.00	0.00	0.00	13.1	2.36	5.31	9.66	0.03	12.3	0.00	0.00	0.00	0.00	0.00	0.00
Dodecanoic acid, ethyl ester	0.06	0.58	0.40	0.08	0.12	0.03	0.03	0.00	0.01	0.00	0.00	31.1	0.04	1.35	0.57	0.01	0.00	0.00	0.00	0.00	0.64	0.00	0.00	0.00	0.00	0.00	0.00	0.00	0.00	0.00	0.00	0.00	0.00	1.41	4.40	2.39	0.00	2.55	0.01	0.00	0.00	0.00	0.00	0.00
Ethyl Acetate	0.00	0.69	2.27	0.08	0.81	0.18	0.15	0.92	0.25	0.21	0.90	6.83	0.03	9.96	12.3	0.00	0.00	0.00	0.00	0.06	1.32	0.00	0.03	0.08	1.28	0.18	0.09	0.00	0.00	3.48	0.00	0.18	11.8	8.80	39.6	4.73	0.03	23.9	0.00	0.04	0.00	0.00	0.12	0.30
Heptane. 2,2,4,6,6-pentamethyl-	0.00	0.00	0.00	0.00	0.07	0.00	0.00	0.00	0.00	0.00	0.00	1.09	0.00	0.00	0.00	0.00	0.02	0.00	0.01	0.05	0.00	0.00	0.00	0.00	0.00	0.00	0.00	0.00	0.07	0.00	0.12	0.00	1.04	0.00	0.00	0.00	0.00	0.00	0.03	0.00	0.01	3.38	0.01	1.46

Hexanoic acid, ethyl ester	0.00	0.09	1.40	0.00	0.06	0.00	0.00	0.00	0.00	0.00	0.47	1.63	0.00	4.32	0.41	0.00	0.00	0.00	0.00	0.00	0.63	0.00	0.00	0.00	0.00	0.00	0.00	0.00	0.00	0.00	0.00	0.00	30.0	0.37	1.57	11.7	0.00	29.9	0.00	0.00	0.00	0.00	0.00	0.00
Hexanoic acid, methyl ester	0.00	0.00	0.00	0.00	0.00	0.00	0.00	0.00	0.00	0.00	0.00	0.00	0.00	0.00	0.47	0.00	0.00	0.00	0.00	0.00	2.32	0.00	0.00	0.00	0.00	0.00	0.00	0.00	0.00	0.00	0.00	0.00	0.00	0.00	0.00	0.82	0.00	0.00	0.00	0.00	0.00	0.00	0.00	0.00
Methyl isovalerate	0.00	0.00	0.00	0.00	0.00	0.00	0.00	0.00	0.00	0.05	0.00	0.00	0.00	0.00	0.00	0.00	0.00	0.00	0.00	0.00	0.00	0.00	0.00	0.00	0.00	0.00	0.00	0.00	0.00	0.00	0.00	0.00	0.00	0.83	3.65	0.91	0.00	0.00	0.00	0.00	0.00	0.00	0.00	0.00
Methyl propionate	0.00	0.00	0.00	0.00	0.00	0.01	0.00	0.00	0.00	0.00	0.00	0.00	0.00	0.00	0.00	0.00	0.00	0.00	0.00	0.00	0.31	0.00	0.00	0.00	0.01	0.00	0.00	0.00	0.00	0.00	0.00	0.00	0.00	2.26	8.83	2.29	0.00	0.23	0.00	0.00	0.00	0.00	0.00	0.00
n-Propyl acetate	0.00	0.02	0.05	0.00	0.00	0.03	0.00	0.00	0.00	0.05	0.00	0.00	0.01	0.13	0.00	0.00	0.00	0.00	0.00	0.00	0.00	0.00	0.00	0.00	1.64	0.00	0.00	0.00	0.00	0.00	0.00	0.00	0.00	7.41	30.1	1.02	0.00	0.00	0.00	0.00	0.00	0.00	0.00	0.03
Octanoic acid, ethyl ester	0.04	0.12	0.52	0.08	0.12	0.00	0.00	0.00	0.00	0.00	0.96	35.4	0.01	5.20	0.00	0.00	0.00	0.00	0.00	0.00	0.75	0.00	0.00	0.00	0.00	0.00	0.00	0.00	0.00	0.00	0.00	0.00	16.9	0.28	1.20	0.83	0.00	13.6	0.00	0.00	0.00	0.00	0.00	0.00
Pentanoic acid, 3-methyl-	0.00	0.00	0.00	0.08	0.31	0.11	0.00	0.00	0.04	0.13	0.55	0.53	2.64	1.31	0.68	0.03	0.03	0.01	0.00	0.00	0.00	0.00	0.00	0.00	0.00	0.00	0.00	0.00	0.00	0.00	0.00	0.00	0.00	0.00	138	0.00	0.00	0.00	0.00	0.00	0.00	0.00	0.00	0.00
Pentanoic acid, 4-methyl-	0.00	0.00	0.00	0.00	0.00	0.00	0.00	0.00	0.00	0.00	0.00	0.00	0.00	0.00	0.00	0.00	0.00	0.00	0.00	0.00	0.00	0.00	0.00	0.00	0.30	0.00	0.00	0.00	0.00	0.00	0.00	0.00	0.94	67.0	263	0.33	0.00	0.00	0.00	0.00	0.00	0.00	0.00	0.00
Pentanoic acid, ethyl ester	0.00	0.00	0.00	0.00	0.00	0.00	0.00	0.00	0.00	0.00	0.00	0.00	0.00	0.00	0.00	0.00	0.00	0.00	0.00	0.00	0.00	0.00	0.00	0.00	0.05	0.00	0.00	0.00	0.00	0.00	0.00	0.00	0.00	1.40	4.47	0.81	0.00	0.00	0.00	0.00	0.00	0.00	0.00	0.00
Propane, 2-(ethenyloxy)-	0.00	0.41	4.09	0.23	0.58	0.02	0.00	0.00	0.00	0.00	0.63	0.00	0.00	1.41	0.31	0.00	0.00	0.00	0.00	0.00	0.15	0.00	0.00	0.00	0.00	0.00	0.00	0.00	0.00	0.00	0.00	0.00	0.00	0.00	0.00	0.00	0.00	0.00	0.00	0.00	0.00	0.00	0.00	0.00
Propanoic acid, 2-methyl-	0.00	0.00	0.00	0.00	0.00	0.00	0.00	0.04	0.00	0.00	0.23	0.00	0.00	0.00	0.00	0.00	0.01	0.00	0.00	0.00	0.00	0.00	0.00	0.00	0.02	0.00	0.00	0.00	0.00	0.00	0.00	0.00	0.00	6.96	26.6	1.47	0.00	0.00	0.00	0.00	0.00	0.00	0.00	0.00
Propanoic acid, 2-methyl-. ethyl ester	0.00	0.26	2.14	0.00	0.08	0.10	0.00	0.00	0.00	0.00	0.00	0.00	0.03	1.51	0.45	0.00	0.00	0.00	0.00	0.00	0.15	0.00	0.00	0.00	0.00	0.00	0.00	0.00	0.00	0.00	0.00	0.00	0.00	2.27	10.2	0.95	0.00	0.00	0.00	0.00	0.00	0.00	0.00	0.00
Propanoic acid, butyl ester	0.00	0.00	0.00	0.00	0.00	0.00	0.00	0.00	0.00	0.00	0.00	0.00	0.00	0.00	0.00	0.00	0.00	0.00	0.00	0.00	0.00	0.00	0.00	0.00	0.00	0.00	0.00	0.00	0.00	0.00	0.00	0.00	0.00	0.00	0.00	2.23	0.00	1.43	0.00	0.00	0.00	0.00	0.00	0.00
Propanoic acid, ethyl ester	0.00	0.00	0.00	0.00	0.00	0.00	0.00	0.00	0.00	0.00	0.00	0.00	0.00	0.07	0.00	0.00	0.00	0.00	0.00	0.00	0.00	0.00	0.00	0.00	0.38	0.00	0.00	0.00	0.00	0.00	0.00	0.00	0.00	19.6	78.8	7.90	0.00	15.4	0.00	0.00	0.00	0.00	0.00	0.00
Propanoic acid, propyl ester	0.00	0.00	0.00	0.00	0.00	0.00	0.00	0.00	0.00	0.00	0.00	0.00	0.00	0.00	0.00	0.00	0.00	0.00	0.00	0.00	0.00	0.00	0.00	0.00	0.39	0.00	0.00	0.00	0.00	0.00	0.00	0.00	0.00	8.75	26.3	2.20	0.00	0.00	0.00	0.00	0.00	0.00	0.00	0.00
Tetradecanoic acid, ethyl ester	0.00	0.00	0.00	0.00	0.00	0.00	0.00	0.00	0.00	0.00	0.00	0.49	0.00	0.00	0.00	0.00	0.00	0.00	0.00	0.00	0.00	0.00	0.00	0.00	0.00	0.00	0.00	0.00	0.00	0.00	0.00	0.00	1.34	0.27	0.72	0.44	0.00	1.02	0.00	0.00	0.00	0.00	0.00	0.00

2-Butanone	0.00	0.00	0.21	0.03	0.13	0.07	0.00	0.17	0.00	0.00	0.00	0.87	0.03	0.00	0.12	0.03	0.01	0.01	0.01	0.00	0.35	0.01	0.00	0.02	0.00	0.04	0.00	0.00	0.01	0.19	0.01	0.02	0.00	0.53	1.25	0.28	0.04	1.24	0.00	0.00	0.00	0.00	2.68	2.43
2-Heptanone	0.00	0.00	1.04	0.29	0.64	0.06	0.00	0.00	0.04	0.02	5.51	0.00	0.00	0.19	1.54	0.00	0.00	0.00	0.00	0.00	1.15	0.00	0.00	0.00	0.00	0.19	0.02	0.00	0.00	0.00	0.00	0.42	9.89	0.00	0.00	1.97	0.00	534	0.01	0.11	0.00	0.00	0.01	0.00
2-Nonanone	0.00	0.00	0.65	0.19	0.50	0.10	0.01	0.00	0.00	0.00	3.87	0.00	0.00	0.13	0.78	0.00	0.00	0.00	0.00	0.00	0.00	0.00	0.00	0.00	0.00	0.08	0.00	0.00	0.06	0.00	0.00	0.29	5.20	0.00	0.00	1.31	0.00	157.7	0.04	0.03	0.00	0.00	0.00	0.00
2-Pentanone	0.00	0.08	0.00	0.00	0.23	0.03	0.03	0.08	0.05	0.00	1.86	0.00	0.02	0.00	0.00	0.00	0.00	0.00	0.00	0.00	0.85	0.00	0.00	0.01	0.00	0.00	0.00	0.00	0.04	0.37	0.00	0.12	0.00	0.00	0.00	0.43	0.00	75.3	0.00	0.00	0.00	0.00	0.00	0.00
2-Undecanone	0.00	0.00	0.29	0.06	0.20	0.03	0.02	0.00	0.00	0.00	0.33	0.00	0.00	0.06	0.00	0.00	0.00	0.00	0.00	0.00	0.00	0.00	0.00	0.00	0.00	0.00	0.00	0.00	0.05	0.00	0.00	0.26	1.68	0.00	0.00	0.42	0.00	2.69	0.00	0.02	0.00	0.00	0.00	0.00
